# Capabilities and limitations of DGGE for the analysis of hydrocarbonoclastic prokaryotic communities directly in environmental samples

**DOI:** 10.1002/mbo3.495

**Published:** 2017-05-18

**Authors:** Dina M. Al‐Mailem, Mayada K. Kansour, Samir S. Radwan

**Affiliations:** ^1^ Microbiology Program Department of Biological Sciences Faculty of Science Kuwait University Safat Kuwait

**Keywords:** Archaea, bacteria, DGGE, environmental samples, molecular analysis

## Abstract

Prokaryotic communities in pristine and oil‐contaminated desert soil, seawater, and hypersaline coastal soil were analyzed using culture‐dependent and culture‐independent approaches. The former technique was the dilution‐plating method. For the latter, total genomic DNA was extracted and the 16S rRNA genes were amplified using a universal bacterial primer pair and primer pairs specific for Actinobacteria, Gammaproteobacteria, and Archaea. The amplicons were resolved using denaturing gradient gel electrophoresis (DGGE) and sequenced, and the sequences were compared to those in GenBank. The plating method offered the advantages of capturing the targeted hydrocarbonoclastic microorganisms, counting them and providing cultures for further study. However, this technique could not capture more than a total of 15 different prokaryotic taxa. Those taxa belonged predominantly to the genera *Alcanivorax, Pseudoxanthomonas, Bosea, Halomonas,* and *Marinobacter*. The individual isolates in culture consumed between 19 and 50% of the available crude oil in 10 days. Although the culture‐independent approach revealed much more microbial diversity, it was not problem‐free. The subdivision primers exhibited satisfactory specificity, but they failed to capture all the available taxa. The universal bacterial primer pair ignored Actinobacteria altogether, although the primer pair specific for Actinobacteria captured many of them, for example, the genera *Geodermatophilus, Streptomyces, Mycobacterium, Pontimonas, Rhodococcus, Blastococcus, Kocuria,* and many others. Because most researchers worldwide use universal primers for PCR, this finding should be considered critically to avoid misleading interpretations.

## 1 INTRODUCTION

Studies in the field of environmental microbiology are confronted with technical problems related to the analysis of microbial communities. At the end of the 19th century, the dilution‐plating method was developed as the pioneer technique for analysis of microbial communities (Koch, 1881). Although since then, many studies have been based on this technique, modern approaches have revealed that microbial diversity is missed by this culture‐dependent approach (Hugenholtz, Goebel, & Pace, 1998). Modern methods are related to metagenomics, that is, the culture‐independent study of genetic material recovered directly from environmental samples (Chen & Pachter, 2005). This approach has been described as “a powerful lens for viewing the microbial world (Marco, 2011).” Confirming the validity of this statement, when we reviewed the literature on hydrocarbonoclastic microorganisms approximately 20 years ago (Radwan & Sorkhoh, 1993), we found a rather limited number of bacterial species recorded worldwide. Our recent reports on hydrocarbonoclastic bacterial species in various ecosystems in Kuwait alone indicate that their numbers have increased dramatically (Al‐Awadhi, Al‐Mailem, Dashti, Hakam, et al., 2012; Al‐Awadhi, Al‐Mailem, Dashti, Khanafer, & Radwan, 2012; Ali, Dashti, Al‐Mailem, Eliyas, & Radwan, 2012; Ali, Dashti, Salamah, Al‐Awadhi, et al., 2016; Ali, Dashti, Salamah, Sorkhoh, et al., 2016; Al‐Mailem, Kansour, & Radwan, 2014a; Al‐Mailem, Kansour, & Radwan, 2014b; Al‐Mailem & Radwan, 2016; Radwan, 2009). Although this improvement is attributed mainly to the use of culture‐independent methods of analysis, practical experience has repeatedly shown that these methods are not problem‐free. Some bias problems associated with using these techniques have been documented in the literature (Al‐Awadhi et al., 2013; Dahllöf, Baillie, & Kjelleberg, 2000; Polz & Cavanaugh, 1998; Reysenbach, Giver, Wickham, & Pace, 1992; Sekiguchi, Tomioka, Nakahara, & Uchiyama, 2001; Sipos et al., 2007; Suzuki & Giovannoni, 1996).

One of the problems is related to the specificity of the primers used for amplification of the 16S rRNA genes. The use of “universal” PCR primers is known to result in ignoring minor constituents of the microbial community because the predominant constituents will prevail in the amplification product. To address this issue, group‐specific PCR primers, for example, for actinobacterial (Stach, Maldonado, Ward, Goodfellow, & Bull, 2003) and gammaproteobacterial (Mühling, Woolven‐Allen, Murrel, & Joint, 2008) 16S rRNA gene fragments, have been developed. However, there are no extensive studies on the specificity strictness of such primers. The major objective of work in this paper, which emphasizes hydrocarbonoclastic microbial communities, is to contribute to filling this information gap. The results are expected to provide researchers in the field of environmental microbiology with useful guidelines for the molecular analysis of microbial communities. It may be argued that the molecular approaches adopted in this study are “old,” while deep sequencing is now the method of choice. While this is certainly true, many laboratories worldwide still use the “old” techniques (e.g., Corsellis, Krasovec, Sylvi, Cuny, & Militon, 2016; Pasumarthi & Mutnuri, 2016; Wu, Dick, Li, & Chen, 2016) and this situation will probably continue in the future. Furthermore, extensive studies are found in the literature, whose interpretation would benefit from the results of this study. One motivation behind this work was to prove that whatever advances in techniques for microbiology analyses would be reached, such techniques will still always have limitations.

## 2 EXPERIMENTAL PROCEDURES

### 2.1 Environmental samples

The environmental samples used in this study represented three ecosystems: a desert soil sample from the Kadma area, north of Kuwait City; a seawater sample from the Arabian Gulf coast at Salmiya, in the middle of Kuwait City; and a hypersaline soil sample from the “sabkha” at the Khiran coast, south of Kuwait. Pristine samples were collected in sterile containers, transported to the laboratory, and subjected to processing on the same day.

Relevant environmental parameters at the sampling sites were measured in triplicate samples taken 20 cm apart using the quality checker WQC‐24, Japan, and APOGEE, USA. These parameters were the pH values; dissolved oxygen content of the seawater; temperature; concentrations of sodium chloride, nitrate, ammonium and phosphate; and total organic carbon content. The oxygen content in soil was measured using an ICTO2 Soil Oxygen Sensor (Armidale, Australia) (Cary & Holder, 1982).

### 2.2 Experimental design

Each of the pristine samples was used for microbiological analysis directly (designated “pristine”) or after having been artificially polluted with crude oil (designated “contaminated”). Aliquots of 50 g of pristine desert soil were suspended in 50 ml aliquots of sterile tap water in screw‐capped 250 ml conical flasks. Portions of seawater (100 ml) were dispensed into 250 ml flasks. Hypersaline coastal soil aliquots, 50 g in conical flasks, were suspended in 50 ml aliquots of hypersaline water (2 mol/L NaCl) from the same site. Contaminated samples were prepared by adding 0.3% w/v light Kuwaiti crude oil to the conical flasks. Flasks in triplicate containing pristine and contaminated samples were incubated on an electrical shaker at 110 rpm and 30°C for 1 month before their contents were analyzed.

### 2.3 Culture‐dependent analysis of hydrocarbonoclastic microorganisms

A selective mineral medium with oil vapor as a sole source of carbon and energy (Sorkhoh, Ghannoum, Ibrahim, Stretton, & Radwan, 1990) was used. Medium aliquots for the analysis of seawater and hypersaline soil were provided with 3% and 12% NaCl, respectively.

Representative colonies were isolated, purified and maintained on the above medium. The colonies were identified by comparing the sequences of their 16S rRNA coding genes with those of strains in the GenBank database, as described previously (Al‐Awadhi et al., 2013).

### 2.4 Determination of oil consumption by individual isolates

Cell suspensions of 1 ml from a common stock (loopful in 10 ml water) of the tested microorganism were inoculated into 250 ml screw‐capped flasks containing 50 ml aliquots of the mineral medium (Sorkhoh et al., 1990) and 0.3% light Kuwaiti crude oil. Control flasks were prepared similarly and autoclaved. Three replicates were prepared throughout. The flasks were inoculated, tightly screw‐capped, and incubated on an electrical shaker at 110 rpm and 30°C for 10 days. Residual hydrocarbons were recovered from each flask with three 20 ml aliquots of pentane. The combined extract was brought to 60 ml with pentane, and 1 μl was analyzed via gas liquid chromatography (GLC) using a Varian 3900 (USA) as described earlier (Al‐Mailem, Eliyas, Khanafer, & Radwan, 2015). The percentage of oil consumption was calculated as the percentage reduction in total hydrocarbon peak area in the GLC profiles based on the total areas of peaks of hydrocarbons recovered from the autoclaved controls.

### 2.5 Culture‐independent analysis of samples using specific primers for amplification

Total genomic DNA was extracted from the sample, purified, and PCR amplified using the four different primer sets as follows: (1) universal primer pair for bacteria (Santegoeds, Ferdelman, Muyzer, & Beer, 1998): GM5F with the sequence 5′‐CCTACGGGAGGCAGCAG‐3′ and 907R with the sequence 5′‐CCGTCAATTCMTTTGAGTTT‐3′; (2) primer pair for Actinobacteria (Heuer, Krsek, Baker, Smalla, & Wellington, 1997): F243 with the sequence GGATGAGCCCGCGGCCTA and R513 with the sequence CGGCCGCGGCTGCTGGCACGTA; (3) primer pair for Gammaproteobacteria (Mühling et al., 2008): Gamma395F with the sequence CMATGCCGCGTGTGTGAA and Gamma871R with the sequence ACTCCCCAGGCGGTCDACTTA; and (4) primer pair for Archaea (Ochsenreiter, Felicitas, & Schleper, 2002): 20F with the sequence TTCCGGTTGATCCYGCCRG and 958R with the sequence YCCGGCGTTGAMTCCAATT.

For denaturing gradient gel electrophoresis (DGGE) analysis, forward primers with GC clamps were used. The acrylamide gel concentration used was 50%–70% for the actinobacterial primer pair and 40%–60% for the remaining three pairs. The DGGE was performed as described previously (Al‐Awadhi et al., 2013). The amplicon bands on the gels were cut out, amplified, and sequenced using the specific primers as described above. The sequences were compared with those of the closest relatives in the GenBank database.

### 2.6 Statistical analysis

Mean readings of the triplicates ± standard deviation values were calculated using Microsoft Excel 2007. The Statistical Package of Social Sciences, version 12, was used to assess the degree of significance, where the *t* test and analysis of variance were used to differentiate between the means of the tested values.

## 3 RESULTS AND DISCUSSION

### 3.1 Environmental parameters

The three ecosystems studied, desert soil, seawater, and hypersaline coastal water, shared the characteristic of being poor in inorganic (nitrate, ammonia, phosphate) and organic carbon nutrients (Table 1). The standard deviation values for the triplicates were <5% of the mean values. However, these three ecosystems varied dramatically in their NaCl contents. In the hypersaline coastal soil, the salinity was significantly (*p *<* *.05) higher than in the seawater ecosystem, in which the salt concentration was significantly (*p *<* *.05) higher than in the desert soil. Consequently, the dissolved oxygen contents were lowest in the hypersaline ecosystem. Oxygen solubility in water decreases with increasing salinity. In this context, the initial step of microbial attack on the hydrocarbon substrate requires molecular oxygen (Ratledge, 1978).

**Table 1:** Environmental parameters at the sampling sites

Parameters	Desert soil	Seawater	Hypersaline coastal soil
pH	7.8 ± 0.1	8.6 ± 0.1	7.2 ± 0.1
Dissolved oxygen (mgl^−1^)	5.9 ± 0.2	5.2 ± 0.1	2.1 ± 0.1
Temperature (°C)	23.0 ± 2.0	20.0 ± 1.0	22.0 ± 2.0
NaCl (%)	1.8 ± 0.1	3.8 ± 0.1	15.2 ± 0.1
Nitrate (mg kg^−1^)	1.6 ± 0.1	1.4 ± 0.1	1.4 ± 0.01
Ammonium(mg kg^−1^)	2.4 ± 0.1	2.2 ± 0.1	2.1 ± 0.01
Phosphate(mg kg^−1^)	1.2 ± 0.1	1.2 ± 0.1	1.4 ± 0.1
Organic carbon (%)	2.4 ± 0.2	2.9 ± 0.2	2.3 ± 0.02

Values were means of 3 determinations ± standard deviation.

### 3.2 Hydrocarbonoclastic microbial communities captured using the culture‐dependent method

The results in Table 2 show that the dilution‐plating method using a mineral medium with oil as a sole source of carbon and energy revealed hydrocarbonoclastic microbial numbers in the magnitude of 10^3^ to 10^7^ colony forming units (CFU) g^−1^. The lowest numbers were counted in the pristine hypersaline coastal soil. However, it was in this extreme ecosystem that oil addition resulted in the highest increases in microbial numbers, from 10^3^ to 10^7^ CFU g^−1^, that is, approximately 10,000‐fold. Increases in numbers of hydrocarbonoclastic microorganisms in response to oil contamination also occurred in the desert and seawater samples but by approximately only two‐ to ninefold. Table 2 also shows the identities and percentage values of the prokaryotic taxa constituting the hydrocarbonoclastic communities. These organisms were affiliated with 15 different “species” only.

**Table 2:** Numbers and identities of hydrocarbonoclastic bacteria isolated from the environmental samples as determined by plating on mineral medium with crude oil vapor as a sole carbon and energy source

Samples	Numbers of CFUs (x 10^4 ^g^−1^)	Isolate identities	% Of the total
Desert soil			
Pristine	20.0 ± 1.8	*Alcanivorax jadensis*	45.5
		*Pseudoxanthomonas wuyuanensis*	41.5
		*Rhizobium rhizoryzae*	8.0
		*Streptomyces graminilatus*	2.5
		*Sinorhizobium meliloti*	2.0
		*Paenibacillus lautus*	0.5
Contaminated	53.0 ± 2.1	*Pseudoxanthomonas wuyuanensis*	66.0
		*Bosea vaviloviae*	44.0
Seawater			
Pristine	3.0 ± 0.02	*Alcanivorax gelatiniphagus*	52.4
		*Alcanivorax venustensis*	46.4
		*Marinobacter algicola*	0.8
		*Mycobacterium chubuense*	0.4
Contaminated	27.0 ± 1.9	*Alcanivorax xenomutans*	55.6
		*Alcanivorax venustensis*	32.0
		*Citreicella marina*	12.3
		*Alteromonas australica*	0.1
Hypersaline coastal soil			
Pristine	0.1	*Halomonas janggokensis*	100.0
Contaminated	1200.0 ± 48.3	*Marinobacter algicola*	100.0

Values were means of 3 determinations ± standard deviation.

Table 3 includes information related to the 16S rRNA gene sequencing of the taxa and to the oil consumption values by those species. The sequence similarities to the GenBank strains were clearly satisfactory, 99%–100%, and all the isolates had a good potential for oil removal.

**Table 3:** Information related to 16S rRNA gene sequencing and oil consumption potential of the hydrocarbonoclastic bacteria isolated from environmental samples

Isolate No. (origin)	Subdivision	Nearest GenBank match	Similarity %	Bases compared	% Oil consumed^a^
1 (PD)	Ac	*Streptomyces graminilatus*	100	342/342	29 ± 1.6
2 (PD)	Al	*Sinorhizobium meliloti*	99	374/375	ND
3 (PD, CD)	Ga	*Pseudoxanthomonas wuyuanensis*	100	430/430	ND
4 (PD)	Al	*Rhizobium rhizoryzae*	99	481/488	50 ± 1.4
5 (PD)	Ba	*Paenibacillus lautus*	100	398/398	30 ± 1.8
6 (CD)	Al	*Bosea vaviloviae*	100	500/500	ND
7 (PD)	Ga	*Alcanivorax jadensis*	100	546/546	25 ± 1.1
8 (CSW)	Ga	*Alteromonas australica*	100	355/355	19 ± 0.9
9 (PS)	Ga	*Halomonas janggokensis*	99	460/461	50 ± 1.8
10 (PSW)	Ga	*Alcanivorax gelatiniphagus*	98	508/517	21 ± 0.8
11 (PSW, CS)	Ga	*Marinobacter algicola*	99	518/525	24 ± 1.0
12 (PSW)	Ac	*Mycobacterium chubuense*	99	458/460	29 ± 1.3
13 (CSW, PSW)	Ga	*Alcanivorax venustensis*	100	546/546	24 ± 1.6
14 (CSW)	Ga	*Alcanivorax xenomutans*	99	487/493	27 ± 1.2
15 (CSW)	Al	*Citreicella marina*	100	434/434	ND

ND, not determined; Ac, Actinobacteria; Al, Alphaproteobacteria; Ga, Gammaproteobacteria; Ba, Bacilli; PD, pristine desert soil; CD, contaminated desert soil; PSW, pristine seawater; CSW, contaminated seawater; PS, pristine hypersaline soil; CS, contaminated hypersaline soil. Sequences were deposited in the GeneBank under the accession numbers KX649774 ‐ KX649788.

a Values were means of three replicates ± standard deviation.

The hydrocarbonoclastic microbial community in the pristine desert soil consisted of 87% Gammaproteobacteria belonging to the genera *Alcanivorax* and *Pseudoxanthomonas*. The remaining 13% were Alphaproteobacteria (10%) belonging to the symbiotic diazotrophic genera *Rhizobium* and *Sinorhizobium*, an actinobacterium (3%) belonging to *Streptomyces* and a bacillus (1%) belonging to *Paenibacillus*. Contaminating this soil with oil resulted in the predominance of the gammaproteobacterium *Pseudoxanthomonas* (66%) and the appearance of the alphaproteobacterium *Bosea vaviloviae* (44%). The hydrocarbonoclastic community in pristine seawater also consisted predominantly (99%) of Gammaproteobacteria belonging to *Alcanivorax* and *Marinobacter* with an actinobacterium, *Mycobacterium* sp., as a minor constituent. In the oil‐contaminated seawater, *Alcanivorax* spp. also predominated and the alphaproteobacterium *Citreicella marina* appeared, making up 12% of the total. In this context, *Alcanivorax* and *Marinobacter* belong to the group of obligate hydrocarbonoclastic bacteria (OHCB) reported to be major contributors to the removal of oil spilled in the marine environment worldwide (Yakimov, Timmis, & Golyshin, 2007). The presence of Alphaproteobacteria known to comprise diazotrophic bacteria (e.g., *Sinorhizobium* and *Rhizobium*; see Table 3) in the communities is interesting in view of the low nitrogen (nitrate and ammonia; Table 1) content of the studied samples. Nitrogen compounds have been reported to be limiting for hydrocarbon biodegradation (Klug & Markovetz, 1971; Leahy & Colwell, 1990). The pristine and contaminated hypersaline coastal samples each contained one gammaproteobacterium, *Halomonas* sp. and *Marinobacter* sp., respectively, but surprisingly no Archaea. In our earlier studies on the same environmental samples, we repeatedly isolated Haloarchaea belonging to *Halobacterium*,* Haloferax*, and other genera (Al‐Mailem, Eliyas, & Radwan, 2014 and Al‐Mailem, Eliyas, et al., 2015). Clearly, the culture‐dependent method did not reveal a satisfactory degree of diversity of the captured hydrocarbonoclastic prokaryotes; it captured a total of only 15 different taxa from the three studied samples.

### 3.3 Microbial communities captured by the culture‐independent method using the universal bacterial primer pair

**Figure 1:**
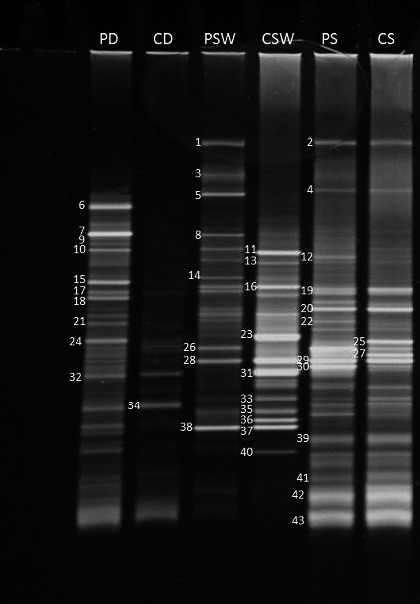
Typical DGGE profile of 16S rDNA amplicons (using universal bacterial primers) in total DNA extracted from desert soil, seawater, and hypersaline soil samples (for band sequencing see Table 4). PD, pristine desert soil; CD, contaminated desert soil; PSW, pristine seawater; CSW, contaminated seawater; PS, pristine hypersaline soil; CS, contaminated hypersaline soil

Figure 1 shows a typical DGGE plate resolving 16S rRNA gene amplicons resulting from the amplification of total genomic DNA extracted from the studied environmental samples using the universal primer. The identities of the amplified bands as determined by comparing their sequences with those in the GenBank database are presented in Table 4.

**Table 4:** Sequencing of the 16S rDNA bands in the DGGE gel, Figure 1

Band No. (origin)	Subdivision	Nearest GenBank match (References citing hydrocarbonoclastic activity)	Similarity %	Bases compared
1 (PSW)	Al	*Candidatus Pelagibacter ubique* (1)	99	470/476
2 (PS)	Fl	*Salinimicrobium gaetbulicola* (1)	99	535/538
3 (PSW)	Al	*Candidatus Pelagibacter ubique* (1)	99	498/502
4 (PS)	Fl	*Salinimicrobium sediminis* (1)	98	531/540
5 (PSW)	Fl	*Lutaonella thermophila*	97	502/518
6 (PD)	Sp	*Chitinophaga ginsengisoli*	98	523/532
7 (PD)	Sp	*Solitalea koreensis* (2)	100	504/504
8 (PSW)	Fl	*Owenweeksia hongkongensis* (2)	99	529/533
9 (PD)	Sp	*Arcticibacter pallidicorallinus*	98	505/515
10 (PD)	Sp	*Solitalea koreensis* (2)	99	492/494
11 (CSW)	Al	*Sphingopyxis granuli* (3)	98	445/452
12 (PS)	Sp	*Aliifodinibius roseus* (4)	99	464/465
13 (CSW)	Ga	*Alcanivorax borkumensis* (5)	98	509/517
14 (PSW)	Al	*Marivita litorea*	98	469/480
15 (PD)	Al	*Brevu*ndim*onas alba* (5)	100	502/502
16 (CSW)	Ga	*Alteromonas australica* (5)	99	529/532
17 (PD)	Al	*Brevundimonas basaltis* (5)	99	500/502
18 (PD)	Al	*Brevundimonas basaltis* (5)	99	496/502
19 (PS)	Ga	*Idiomarina piscisalsi* (6)	98	529/542
20 (PS)	Ga	*Idiomarina zobellii* (6)	99	503/505
21 (PD)	Al	*Porphyrobacter tepidarius* (7)	99	503/507
22 (PS)	Sp	*Aliifodinibius roseus* (4)	99	516/517
23 (CSW)	Al	*Hyphomonas atlantica* (8)	97	500/515
24 (PD)	Al	*Porphyrobacter neustonensis* (7)	99	518/520
25 (CS)	Al	*Porphyrobacter neustonensis* (7)	99	528/530
26 (PSW)	Sp	*Aliifodinibius roseus* (4)	100	545/545
27 (CS)	Sp	*Aliifodinibius roseus* (4)	99	502/503
28 (PSW)	Ga	*Marinobacter lipolyticus* (5)	99	520/526
29 (PS)	Sp	*Aliifodinibius roseus* (4)	99	519/521
30 (PS)	Sp	*Aliifodinibius roseus* (4)	100	496/496
31 (CSW)	Al	*Thalassospira australica* (9)	100	520/520
32 (PD)	Al	*Sphingomonas sediminicola* (5)	99	514/518
33 (CSW)	Al	*Maricaulis parjimensis* (10)	100	515/515
34 (CD)	Al	*Phenylobacterium panacis* (11)	100	521/521
35 (CSW)	Ga	*Alcanivorax marinus* (5)	99	498/505
36 (CSW)	Ph	*Algisphaera eraagarilytica*	99	414/420
37 (CSW)	Ga	*Alcanivorax marinus* (5)	100	530/530
38 (PSW)	Ga	*Alcanivorax dieselolei* (5)	100	551/551
39 (PS)	Bac	*Salinibacter ruber* (12)	97	521/536
40 (CSW)	Ph	*Phycisphaera mikurensis*	98	507/520
41 (PS)	Ha	*Salarchaeum japonicum* (13)	98	494/505
42 (PS)	Ha	*Halomicrobium zhouii* (14)	99	466/468
43 (PS)	Ha	*Halorhabdus tiamatea* (12)	99	475/477

PD, pristine desert soil; CD, contaminated desert soil; PSW, pristine seawater; CSW, contaminated seawater; PS, pristine hypersaline soil; CS, contaminated hypersaline soil; Ac, Actinobacteria; Al, Alphaproteobacteria; Ga, Gammaproteobacteria; Fl, Flavobacteriia; Sp, Sphingobacteriia; Ph, Phycisphaerae; Bac, Bacteroidetes; Ha, Halobacteria. Sequences were deposited in the GeneBank under the accession numbers KX649789 ‐ KX649831. References: (1) Al‐Awadhi et al. (2013); (2) Al‐Mailem et al. (2014a); (3) LaRoe, Wang, & Han (2010); (4) Al‐Mailem, Eliyas, et al. (2015); (5) Al‐Awadhi, Al‐Mailem, Dashti, Hakam, et al. (2012); (6) Radwan, Mahmoud, Khanafer, Al‐Habib, & Al‐Hasan (2010); (7) Gauthier et al. (2003); (8) Kappell et al. (2014); (9) Ali, Dashti, Salamah, Al‐Awadhi, et al. (2016); (10) Vila, Nieto, Mertens, Springael, & Grifoll (2010); (11) Ali, Dashti, Salamah, Sorkhoh, et al. (2016); (12) Corsellis et al. (2016); (13) Al‐Mailem, Kansour, & Radwan (2015); (14) Cui et al. (2009).

In total, 43 bands in the six environmental samples were successfully sequenced. Given that in addition, approximately as many bands failed to be sequenced, it is clear how powerful this molecular approach is in revealing microbial diversity. To review, the culture‐dependent method captured only 15 taxa. The latter technique is selective in capturing only hydrocarbonoclastic microorganisms. Nevertheless, the difference in efficiency of revealing diversity among microbial communities between the two methods is clear.

The use of a universal primer pair in amplification promises the capture of all bacterial subdivisions. The results in Table 4 reveal that this potential was fulfilled but only to some extent. A total of seven subdivisions are shown in this table, and the dominant ones were the Alphaproteobacteria and Gammaproteobacteria. Both subdivisions, especially the Alphaproteobacteria, comprised aquatic gram‐negative bacteria with oligotrophic and diazotrophic taxa. However, it is noteworthy and surprising that the primer pair ignored Actinobacteria and Bacilli, although the culture‐dependent approach, with its extremely limited capturing capacity, revealed two Actinobacteria and one taxon belonging to Bacilli in the studied samples (Tables 2 and 3). It will be shown below that all the studied samples were rather rich in taxa belonging to this subdivision when the actinobacterial primer pair was used for amplification.

An interesting result is that the oil‐contaminated samples always contained fewer bands than the pristine samples. It is possible that oil enriches hydrocarbonoclastic members at the expense of the nonhydrocarbonoclastic ones. Among the few bands enriched by oil, was band 34 of *Phenylobacterium*, which is hydrocarbonoclastic (Ali, Dashti, Salamah, Sorkhoh, et al., 2016). The desert soil samples revealed only Alphaproteobacteria and Sphingobacteriia; the seawater samples contained Alphaproteobacteria, Gammaproteobacteria, Flavobacteriia, Sphingobacteriia, and Phycisphaerae; and the hypersaline coastal soil contained Sphingobacteriia, halobacteria, Gammaproteobacteria, Flavobacteriia, Alphaproteobacteria, and Bacteroidetes. Taxa in seawater that were enriched by oil contamination, as tentatively judged by the densities of their gene bands, were affiliated with *Sphingopyxis granuli* [band 11], *Alcanivorax borkumensis* [13], *Alteromonas australica* [16], *Hyphomonas atlantica* [23], *Marinobacter lipolyticus* [28], *Thalassospira australica* [31], *Maricaulis parjimensis* [33], *Alcanivorax marinus* [35, 37], *Algisphaera agarilytica* [36], and *Phycisphaera mikurensis* [40], most of which are prominent hydrocarbon utilizers. In the oily hypersaline soils, *Aliifodinibius roseus* [26, 27], *Salinibacter ruber* [39], and *Salarchaeum japonicum* [41] were enriched, although both the contaminated and pristine samples contained hydrocarbonoclastic taxa, for example, *Idiomarina* spp. [19, 20]. Comparing the results in Table 4 with those in Table 2 clearly shows that the culture‐independent method was much more powerful in capturing diverse bacterial taxa than the culture‐dependent method. As the culture‐independent approach failed to provide information of the hydrocarbonoclastic potential of the taxa, we cited for the microbial names in Table 4 the pertinent references in the available literature that confirm the probable hydrocarbonoclastic nature of the respective organisms. Needless to say, several of the other yet‐unstudied taxa may also contain hydrocarbonoclastic activity.

### 3.4 Microbial communities captured by the culture‐independent method using the actinobacterial primer pair

**Figure 2:**
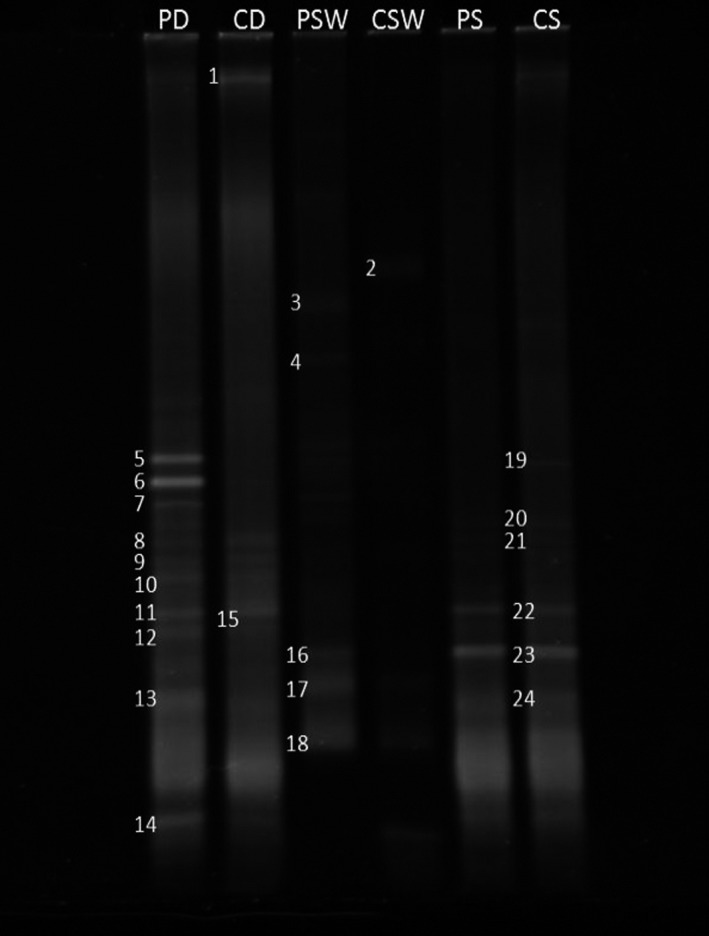
Typical DGGE profile of 16S rDNA amplicons (using Actinobacterial‐specific primers) in total DNA extracted from desert soil, seawater, and hypersaline soil samples (for band sequencing see Table 5). PD, pristine desert soil; CD, contaminated desert soil; PSW, pristine seawater; CSW, contaminated seawater; PS, pristine hypersaline soil; CS, contaminated hypersaline soil

Figure 2 shows a typical DGGE plate resolving 16S rRNA genes resulting from the amplification of total genomic DNA extracts from the studied environmental samples using the specific actinobacterial primer pair. The identities of amplified bands from this gel are provided in Table 5.

**Table 5:** Sequencing of the 16S rDNA bands in the DGGE gel, Figure 2

Band No. (origin)	Subdivision	Nearest GenBank match (References citing hydrocarbonoclastic activity)	Similarity %	Bases compared
1 (CD)	Ac	*Geodermatophilus terrae* (1)	99	206/207
2 (CSW)	Ac	*Mycobacterium europaeum* (2)	100	181/181
3 (PSW)	Ac	*Streptomyces chiangmaiensis* (3)	99	247/251
4 (PSW)	Ac	*Streptomyces glaucescens* (3)	97	200/206
5 (PD)	Ac	*Pontimonas salivibrio*	99	188/190
6 (PD)	Ac	*Pontimonas salivibrio*	97	220/226
7 (PD)	Ac	*Saccharopolyspora acebuensis*	97	233/239
8 (PD)	Ac	*Geodermatophilus terrae* (1)	98	201/205
9 (PD)	Ac	*Actinokineospora bangkokensis*	99	254/257
10 (PD)	Ac	*Blastococcus jejuensis*	99	204/205
11 (PD)	Ac	*Kineosphaera nakaumiensis*	99	200/203
12 (PD)	Ac	*Rhodococcus maanshanensis* (3)	99	205/208
13 (PD)	Ac	*Kribbella italica* (4)	97	225/231
14 (PD)	Ac	*Blastococcus jejuensis*	100	208/208
15 (CD)	Ac	*Streptomyces racemochromogenes* (3)	98	244/249
16 (PSW)	Ac	*Streptomyces glaucescens* (3)	99	211/214
17 (PSW)	Ac	*Tetrasphaera australiensis*	98	211/215
18 (PSW)	Ac	*Tetrasphaera australiensis*	100	247/247
19 (CS)	Ac	*Actinocatenispora sera*	99	200/203
20 (CS)	Ac	*Geodermatophilus tzadiensis* (1)	99	204/205
21 (CS)	Ac	*Rhodococcus agglutinans* strain (3)	99	198/200
22 (CS)	Ac	*Kocuria dechangensis* (5)	99	250/252
23 (CS)	Ac	*Streptomyces sundarbansensis* (3)	98	242/248
24 (CS)	Ac	*Saccharothrix mutabilis* (6)	98	208/212

PD, pristine desert soil; CD, contaminated desert soil; PSW, pristine seawater; CSW, contaminated seawater; PS, pristine hypersaline soil; CS, contaminated hypersaline soil; Ac, Actinobacteria. Sequences were deposited in the GeneBank under the accession numbers KX649832 ‐ KX649855. References: (1) Wu et al. (2016); (2) Al‐Mailem et al. (2014b); (3) Al‐Awadhi, Al‐Mailem, Dashti, Hakam, et al. (2012); (4) Chen, Vohra, & Murrell (2010); (5) Al‐Mailem, Eliyas, et al. (2015); (6) Hu, Ren, Zhou, Xia, & Liu (2003).

A total of 24 amplicon bands were successfully sequenced. In addition, several other bands failed to be sequenced. The primer pair exclusively amplified Actinobacteria. While this could be considered a point of strength of the culture‐independent approach, it is also a point of weakness. The richness of actinobacterial species in Table 5 compared with their absence in Table 4 shows that depending on a universal bacterial primer pair alone, as frequently reported in the literature, leads to ignoring many actinobacterial species actually present in the same environmental samples. Members of this subdivision captured in the desert soil were affiliated with the genera *Geodermatophilus* [bands 1, 8], *Pontimonas* [5, 6], *Saccharopolyspora* [7], *Actinokineospora* [9], *Blastococcus* [10], *Kineosphaera* [11], *Rhodococcus* [12], *Kribbella* [13], *Blastococcus* [14], and *Streptomyces* [15]. The latter taxon was enriched in the oil‐contaminated desert soil, as tentatively indicated by the band density. Actinobacterial genera identified in the seawater were *Mycobacterium* [band 2]*, Streptomyces* [3, 4, 16], and *Tetrasphaera* [17]; the former was especially enriched in response to oil addition. Actinobacterial genera in the hypersaline coastal soil were affiliated with *Actinocatenispora* [band 19]*, Geodermatophilus* [20], *Rhodococcus* [21]*, Kocuria* [22], *Streptomyces* [23], and *Saccharothrix* [24]. As shown in Table 5, many of the taxa listed above were reported earlier as hydrocarbonoclastic. It is also clear that fewer bands were present in oily than in pristine samples. The former were apparently those of bacteria with high hydrocarbonoclastic potential. The two species belonging to the two genera, *Streptomyces* and *Mycobacterium,* captured by the culture‐dependent method (Table 2) were different from the species captured by the culture‐independent method.

### 3.5 Microbial communities captured by the culture‐independent method using gammaproteobacterial primers

**Figure 3:**
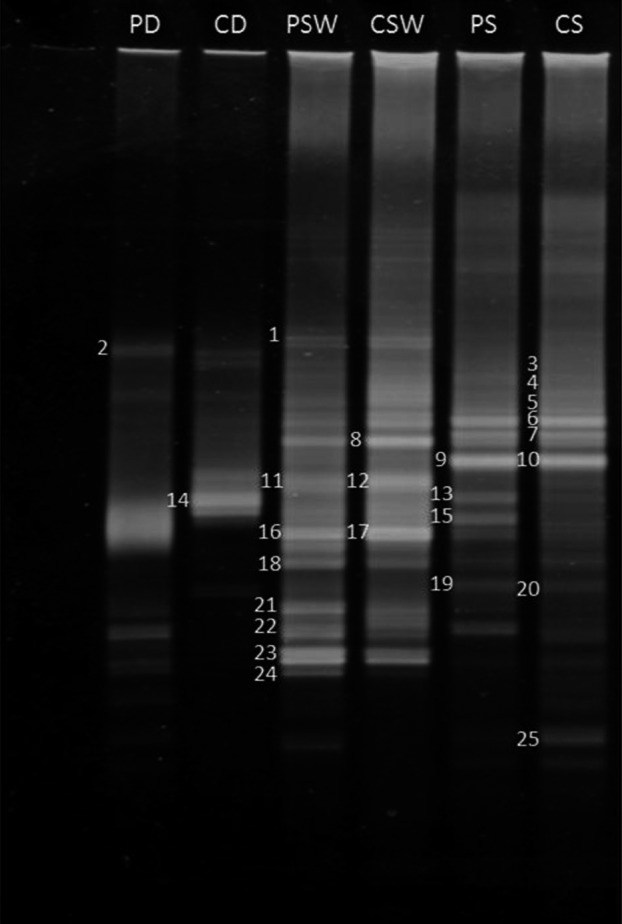
Typical DGGE profile of 16S rDNA amplicons (using Gammaproteobacteria‐specific primers) in total DNA extracted from desert soil, seawater, and hypersaline soil samples (for band sequencing see Table 6). PD, pristine desert soil; CD, contaminated desert soil; PSW, pristine seawater; CSW, contaminated seawater; PS, pristine hypersaline soil; CS, contaminated hypersaline soil

Figure 3 illustrates a typical DGGE plate resolving 16S rRNA genes resulting from amplification of total genomic DNA extracted from the studied experimental samples using the specific gammaproteobacterial primer pair. The identities of amplified bands on this gel are provided in Table 6.

**Table 6:** Sequencing of the 16S rDNA bands in the DGGE gel, Figure 3

Band No. (origin)	Subdivision	Nearest GenBank match (References citing hydrocarbonoclastic activity)	Similarity %	Bases compared
1 (PSW)	Ga	*Marinobacter nitratireducens* (1)	99	459/461
2 (PD)	Ga	*Cellvibrio fulvus*	100	461/461
3 (CS)	Ga	*Idiomarina seosinensis* (2)	99	431/432
4 (CS)	Ga	*Idiomarina seosinensis* (2)	98	440/447
5 (CS)	Ga	*Idiomarina seosinensis* (2)	99	451/452
6 (CS)	Ga	*Idiomarina seosinensis* (2)	99	453/455
7 (CS)	Ga	*Idiomarinas eosinensis* (2)	100	457/457
8 (CSW)	Ga	*Aestuariibacter halophilus* (3)	99	446/449
9 (PS)	Ga	*Idiomarina seosinensis* (2)	100	466/466
10 (CS)	Ga	*Idiomarina zobellii* (2)	100	445/445
11 (PSW)	Ga	*Marinobacter nitratireducens* (1)	97	382/392
12 (CSW)	Ga	*Marinobacter lipolyticus* (1)	98	450/457
13 (PS)	Ga	*Marinobacter zhanjiangensis* (1)	100	402/402
14 (CD)	Ga	*Pseudomonas songnenensis* (4)	100	464/464
15 (PS)	Ga	*Marinimicrobium locisalis*	99	452/454
16 (PSW)	Ga	*Marinobacter goseongensis* (1)	99	442/444
17 (CSW)	Ga	*Marinobacter lipolyticus* (1)	99	438/444
18 (PSW)	Ga	*Alcanivorax dieselolei* (1)	99	456/458
19 (PS)	Ga	*Gilvimarinus polysaccharolyticus*	98	446/453
20 (CS)	Ga	*Marinimicrobium haloxylanilyticum*	99	397/403
21 (PSW)	Ga	*Alcanivorax venustensis* (1)	100	460/460
22 (PSW)	Ga	*Alcanivorax venustensis* (1)	100	456/456
23 (PSW)	Ga	*Alcanivorax venustensis* (1)	100	458/458
24 (PSW)	Ga	*Alcanivorax venustensis* (1)	100	450/450
25 (CS)	Ga	*Halomonas janggokensis* (5)	99	442/444

PD, pristine desert soil; CD, contaminated desert soil; PSW, pristine seawater; CSW, contaminated seawater; PS, pristine hypersaline soil; CS, contaminated hypersaline soil; Ga, Gammaproteobacteria. Sequences were deposited in the GeneBank under the accession numbers KX649856 ‐ KX649880. References: (1) Al‐Awadhi, Al‐Mailem, Dashti, Hakam, et al. (2012); (2) Radwan et al. (2010); (3) Wang, Zhang, Shan, & Shao (2014); (4) Al‐Mailem et al. (2014b); (5) Al‐Mailem, Eliyas, et al. (2014).

As many as 25 bands were successfully sequenced and identified. Several other bands failed to be sequenced. This relatively large number of amplicon bands reflects the diversity of the gammaproteobacterial taxa in the microbial communities. As should be expected, many more gammaproteobacterial bands were found in the seawater and hypersaline coastal soil than in the desert soil.

The primer pair exclusively amplified Gammaproteobacteria (Table 6). The desert soil harbored only two genera belonging to this subdivision: *Cellvibrio* [band 2] in the pristine and *Pseudomonas* [14] in the oil‐contaminated samples. Neither of the two was captured by the culture‐dependent method (Table 2) or by the culture‐independent method using the universal bacterial primer pair, although the two techniques captured other genera belonging to the Gammaproteobacteria. The seawater samples contained most of the gammaproteobacterial gene bands that were affiliated with the genera *Marinobacter* [bands 1, 12, 16, 17]*, Alcanivorax* [18, 21‐24], and *Aestuariibacter* [8], the former and latter of which were enriched in the oil‐contaminated water. As in many previous reports, *M. nitratireducens, M. lipolyticus,* and *A. venustensis* exhibited multiple bands on the gel. The hypersaline coastal soil contained Gammaproteobacteria affiliated predominantly with the genera *Idiomarina* [bands 3‐7, 9, 10, which exhibited multiple bands], *Marinimicrobium* [15]*, Marinobacter* [13]*, Gilvimarinus* [15], and *Halomonas* [25]. In the oil‐contaminated samples, *Idiomarina, Marinimicrobium,* and *Halomonas* were enriched. Of these genera, only *Marinobacter, Alcanivorax,* and *Halomonas* were captured by the culture‐dependent method and *Idiomarina, Marinobacter,* and *Alcanivorax* by the culture‐independent method using the universal primers for amplification.

### 3.6 Microbial communities captured by the culture‐independent method using the archaeal primer pair

**Figure 4:**
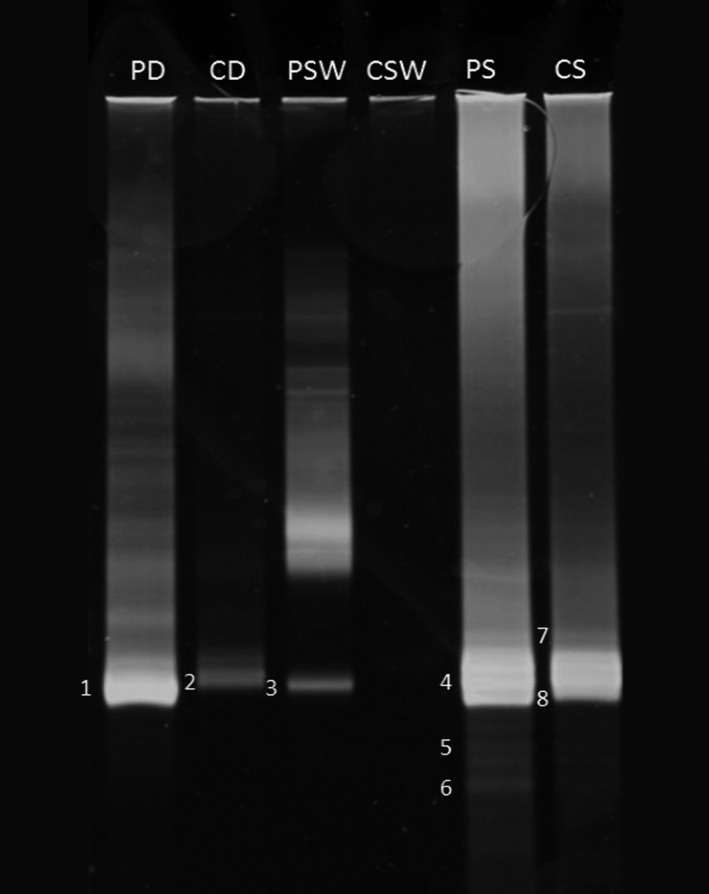
Typical DGGE profile of 16S rDNA amplicons (using archaeal primers) in total DNA extracted from desert soil, seawater, and hypersaline soil samples (for band sequencing see Table 7). PD, pristine desert soil; CD, contaminated desert soil; PSW, pristine seawater; CSW, contaminated seawater; PS, pristine hypersaline soil; CS, contaminated hypersaline soil

Figure 4 shows the resolution of 16S rRNA genes resulting from the amplification of total genomic DNA extracted from the samples using the archaeal primer pair for amplification. The identities of amplified bands on this gel are available in Table 7.

**Table 7:** Sequencing of the 16S rDNA bands in the DGGE gel, Figure 4

Band No. (origin)	Subdivision	Nearest GenBank match (References citing hydrocarbonoclastic activity)	Similarity %	Bases compared
1 (PD)	Ni	*Nitrososphaera viennensis*	99	832/839
2 (CD)	Ni	*Nitrososphaera viennensis*	99	830/840
3 (PSW)	Th	*Methanomassiliicoccus luminyensis*	99	837/844
4 (PS)	Ha	*Halosimplex carlsbadense* (1)	99	859/864
5 (PS)	Ha	*Halomicrobium zhouii* (2)	100	847/847
6 (PS)	Ha	*Halorhabdus tiamatea*	99	840/845
7 (CS)	Ha	*Halosimplex pelagicum* (1)	99	777/782
8 (CS)	Ha	*Salinirubrum litoreum*	99	847/856

PD, pristine desert soil; CD, contaminated desert soil; PSW, pristine seawater; CSW, contaminated seawater; PS, pristine hypersaline soil; CS, contaminated hypersaline soil; Ni, Nitrososphaeria; Th, Thermoplasmata; Ha, Halobacteria. Sequences were deposited in the GeneBank under the accession numbers KX649881 ‐ KX649888. References: (1) Dalvi, Youssef, & Fathepure (2016); (2) Cui et al. (2009).

Only eight bands were successfully sequenced and identified. However, this primer pair also fulfilled the specificity promise, as only archaeal taxa were amplified. *Nitrososphaera viennensis* [bands 1, 2] was found in the desert soil, and *Methanomassiliicoccus luminyensis* [3] was identified in the pristine seawater. As expected, most of the Archaea were halobacteria, belonging to the genera *Halosimplex* [4]*, Halomicrobium* [5]*, Halorhabdus* [6, 7], and *Salinirubrum* [8]. None of these Archaea had been captured by the culture‐dependent method, but *Halomicrobium* and *Halorhabdus* had been captured by the culture‐independent method using the universal primer pair for amplification. *Halosimplex pelagicum* and *Salinirubrum litoreum* were enriched in the oil‐contaminated hypersaline coastal sample. It is surprising that neither of the typical oil‐utilizing archaeal genera *Halobacterium* and *Haloferax,* which we repeatedly found in samples from the same hypersaline coastal region (Al‐Mailem, Eliyas, et al., 2015; Al‐Mailem, Eliyas, et al., 2014), appeared in this analysis. The reason may be the relatively low salt content of the hypersaline coastal area following precipitation just before sampling; the NaCl content during a dry summer may exceed 25%, which normally enhances the latter Archaea.

## 4 CONCLUSIONS

Technical bias problems related to the molecular analysis of microbial communities in environmental samples are well documented. Such problems may be due to template annealing in the amplification of 16S rRNA genes (Suzuki & Giovannoni, 1996), template‐to‐product ratios in multi‐template PCR (Polz & Cavanaugh, 1998), limitations inherent in 16S rRNA gene interspecies heterogeneity (Dahllöf et al., 2000), single DGGE bands not always representing single bacterial strains (Sekiguchi et al., 2001), primer mismatch, annealing temperature, and PCR cycle numbers affecting the 16S rRNA gene‐targeting (Sipos et al., 2007), intraspecific polymorphism of 16S rRNA genes (Cui, Zhou, Oren, & Liu, 2009) and differential 16S rRNA gene amplification by primers (Al‐Awadhi et al., 2013). The novelty of this study is that it sheds light on additional bias problems other than those reviewed above. Using new environmental samples, we confirmed here our recent finding (Al‐Awadhi et al., 2013) that the frequently used bacterial universal primers ignore the major subdivision of Actinobacteria, although representatives of this subdivision may actually be quite frequent in the studied samples. Neglecting this fact would lead to misinterpretation of old and new findings on microbial community composition in environmental samples.

It was not the major objective of this study to discuss the frequency of hydrocarbonoclastic prokaryotes in relation to the prevailing environmental parameters. Although the major objective was to evaluate common techniques used in the microbial analysis of environmental samples, the former subject could not be completely neglected. One conclusion of this paper is that for a comprehensive view of the microbial communities of environmental samples, a culture‐dependent method should be adopted along with the culture‐independent approach using primers specific for various subdivisions. Each individual analysis gives its own unique list of microorganisms. The collective result would provide a microbial community structure closest to reality, as suggested previously (Shade et al., 2012).

It may be argued that hydrocarbonoclastic microorganisms in environmental samples could have been analyzed using alkane‐specific primers. However, experience in our laboratory revealed repeatedly that the *alkB* genes frequently failed to be demonstrated even in classic alkane degraders, when some of the commercially available primers were used. Earlier researchers encountered similar experience (Heiss‐Blanquet, Benoit, Maréchaux, & Monot, 2005; Jurelevicius, Alvarez, Peixoto, Rosado, & Seldin, 2013; Kloos, Munch, & Schloter, 2006).

## CONFLICT OF INTEREST

None declared.
